# White matter microstructural damage and its effect on cognitive impairment in patients with metabolic syndrome and cerebral small vessel disease

**DOI:** 10.3389/fneur.2025.1698280

**Published:** 2026-01-16

**Authors:** Han Liu, He Feng, Bo Tian, Kaigui Wang, Yali Chen, Xia Zhou, Wenhui Zheng, Wei Zhang, Yu Xia, Daping Lv, Xiaoqun Zhu, Zhongwu Sun

**Affiliations:** 1 Department of Neurology, The First Affiliated Hospital of Anhui Medical University, Hefei, China; 2 Department of Neurology, Anhui Public Health Clinical Center, Hefei, China; 3 Department of Neurology, The Third People's Hospital of Hefei, Hefei, China

**Keywords:** cerebral small vessel disease, cognitive impairment, diffusion tensor imaging, metabolic syndrome, white matter

## Abstract

**Background:**

Metabolic syndrome (MetS) is linked to cerebral small vessel disease (CSVD) and cognitive impairment in the elderly, but the underlying mechanisms remain unclear. This study investigated whether white matter (WM) microstructural damage mediates the relationship between CSVD severity and cognitive function in patients with MetS.

**Methods:**

A total of 170 right-handed participants aged 50–80 years were recruited, including 75 with MetS and 95 healthy controls (HC). MetS participants were divided into MetS without CSVD (MetS-NCSVD, *n* = 22) and MetS with CSVD (MetS-CSVD, *n* = 53). Cognitive function was assessed using Montreal cognitive assessment, Stroop color word test (SCWT), trail making test (TMT) and geriatric depression scale. WM integrity was evaluated using diffusion tensor imaging, including fractional anisotropy (FA) and mean diffusivity (MD). Mediation analysis was performed to examine the role of WM microstructural damage in the relationship between CSVD severity and cognitive impairment.

**Results:**

The MetS-CSVD group had significantly lower FA and higher MD in core WM fiber tracts compared to MetS-NCSVD and HC groups (*P* < 0.001). In the MetS group, FA in these tracts were inversely correlated with cognitive test scores (TMT-B and SCWT-C, *P* < 0.001). Multiple linear regression analyses confirmed these associations (*P* ≤ 0.045). Mediation analysis revealed that FA values in WM fiber tracts may mediate the relationship between CSVD severity and cognitive function.

**Conclusions:**

Our findings suggest that WM microstructural damage may account for the association between CSVD and cognitive impairment, acting as a potential mediator in MetS patients. Future longitudinal studies are needed to validate these findings and explore causal mechanisms.

## 1 Introduction

Metabolic syndrome (MetS) is a well-recognized cluster of cardiovascular risk factors, including central obesity, elevated triglycerides (TG), low high-density lipoprotein cholesterol (HDL-C), hypertension, and hyperglycemia (1). A diagnosis of MetS requires the presence of at least three of these five criteria (2). Identified as a global health concern by the World Health Organization, MetS is associated with a significantly elevated risk of atherosclerosis and cardiovascular disease, contributing to increased morbidity and mortality (3). Epidemiological studies have demonstrated that individuals with MetS exhibit a 1.5-fold increased risk of cardiovascular disease and a 2-fold higher risk of stroke-related mortality (4). In addition to its cardiovascular implications, MetS is also associated with an increased risk of vascular dementia (VaD) (5). Population-based studies and meta-analyses have shown that individuals with MetS are more likely to develop all-cause dementia and VaD compared to those without MetS (6, 7). Both cross-sectional and long-term longitudinal studies have further revealed a link between MetS and cognitive impairment (8, 9).

Cerebral small vessel disease (CSVD) is a major underlying cause of vascular cognitive impairment (10, 11). CSVD is characterized by neuroimaging markers, such as white matter hyperintensities (WMH), microinfarcts, lacunar infarcts, and enlarged perivascular spaces, all of which contribute to structural brain damage and cognitive impairment (12, 13). MetS is hypothesized to accelerate the development and progression of CSVD by inducing vascular dysfunction, leading to chronic microvascular injury, white matter (WM) microstructural damage, and brain atrophy (14). The individual components of MetS, including hypertension, hyperglycemia, and dyslipidemia, may independently contribute to cerebral small vessel pathology (15). Several studies have explored the associations between hypertension, type 2 diabetes mellitus (T2DM), and CSVD, demonstrating that CSVD is a key mediator linking these metabolic disturbances to cognitive impairment (16, 17). A potential mechanism underlying this relationship is the disruption of WM integrity due to chronic vascular dysfunction, a hypothesis supported by growing evidence of an association between MetS and WM microstructural alterations in the brain (14, 18, 19).

However, the extent to which MetS contributes to CSVD-related cognitive impairment remains unclear. Moreover, the specific role of WM microstructural alterations in mediating the relationship between CSVD severity and cognitive impairment in the context of MetS has not been fully elucidated. To address this gap, we will investigate whether MetS exacerbates CSVD-related WM microstructural damage and cognitive impairment by comparing participants with MetS and CSVD (MetS-CSVD), those with MetS but without CSVD (MetS-NCSVD), and healthy controls (HC). Using diffusion tensor imaging (DTI), which provides quantitative measures of WM integrity by assessing the diffusion properties of water molecules in neural tissues (20), we will explore whether WM microstructural alterations mediate the relationship between CSVD severity and cognitive impairment. The microstructural integrity will be assessed through two principal DTI metrics, fractional anisotropy (FA) and mean diffusivity (MD). FA reflects the directionality of water diffusion and serves as a marker of axonal integrity and myelination, whereas MD represents the overall magnitude of diffusion, which increases with tissue disruption (21). These metrics are highly sensitive to the subtle WM microstructural damage characteristic of both CSVD and MetS (21, 22). We hypothesize that MetS may exacerbate CSVD-related cognitive impairment through WM microstructural damage, and this study aims to provide novel insights into the underlying mechanisms.

## 2 Methods

### 2.1 Study participants

A total of 170 right-handed participants aged 50–80 years were recruited from the First Affiliated Hospital of Anhui Medical University. Participants were included if they met ≥3 of the following MetS criteria: (1) central obesity (waist circumference [WC] of ≥ 90 cm for men, and ≥ 80 cm for women); (2) elevated TG (≥1.7 mmol/L); (3) low HDL-C ( ≤ 1.03 mmol/L for men, 1.3 mmol/L for women); (4) hypertension (blood pressure ≥130/85 mmHg or the use of anti-hypertensive medication); and (5) hyperglycemia (fasting blood glucose [FBG] ≥ 5.6 mmol/L or the use of anti-glycemic medication) (2). 75 participants diagnosed with MetS were assigned to the MetS group. The MetS participants were further stratified into two subgroups based on the presence of CSVD: MetS with CSVD (MetS-CSVD) and MetS without CSVD (MetS-NCSVD). The inclusion criteria for CSVD were as follows: (1) subcortical WMH with a Fazekas score of ≥ 2 or periventricular WMH with a Fazekas score of 3; (2) cerebral microbleeds (CMB) detected as signal voids (3–15 mm) on T2^*^-weighted angiographic sequences, or low-signal lesions (2–10 mm) on susceptibility-weighted imaging (SWI) (23). Exclusion criteria included specific causes for WM lesions (e.g., multiple sclerosis, brain radiation), neurodegenerative diseases (e.g., Alzheimer's disease, Parkinson's disease), psychiatric disorders, alcohol or drug abuse, magnetic resonance imaging (MRI) contraindications (e.g., ferromagnetic implants, stents, claustrophobia), cerebral hemorrhage, history of brain trauma, brain tumor or space-occupying lesion, and cerebral infarction with a diameter of ≥20 mm on MRI. Furthermore, 95 participants, who did not meet the diagnostic criteria for MetS or CSVD and were comparable in age, sex, and years of education, were enrolled as the HC group.

The study was approved by the Ethics Committee of the First Affiliated Hospital of Anhui Medical University (Ethics Approval Number: Quick-PJ 2023-01-29). Written informed consent was obtained from all participants prior to enrollment.

### 2.2 Risk factor measurement

Fasting blood samples were collected to determine TG, HDL-C, and FBG levels. Blood pressure was measured using a standard sphygmomanometer after 5 min of rest, with a second measurement taken after another 5 min and the average recorded. WC was measured at the umbilical level while participants stood. Medications for hypertension, diabetes, or cholesterol abnormalities were also recorded.

### 2.3 Neuropsychological assessment

Researchers conducting the neuropsychological evaluations were blinded to participant grouping. Cognitive function was assessed using the Montreal cognitive assessment (MoCA). Executive function, attention, visual spatial abilities, and information processing speed were evaluated with the Stroop color word test (SCWT, including SCWT-A for dots, SCWT-B for words, and SCWT-C for color words) and the trail making test (TMT-A and TMT-B). Mood disorders were assessed using the geriatric depression scale (GDS). Tests were administered in randomized order during a single, ~2-h session.

### 2.4 MRI data acquisition

MRI data were acquired using a 3.0 Tesla MRI system (Discovery MR750w, General Electric, Milwaukee, WI, USA) equipped with a 24-channel head coil. High-resolution 3D T1-weighted brain scans were performed for anatomical reference and tissue segmentation with the following parameters: repetition time (TR) = 8.5 ms, flip angle = 12°, echo time (TE) = 3.2 ms, matrix size = 256 × 256, field of view (FOV) = 256 × 256 mm^2^, slice thickness = 1 mm (no gap), voxel size = 1 × 1 × 1 mm^3^, 188 sagittal sections. T2 Fluid-attenuated inversion recovery (FLAIR) sequences (TE = 119.84 ms, TR = 9000 ms, flip angle = 90°, matrix = 512 × 512, FOV = 225 × 225 mm^2^, 19 slices × 7.0 mm thickness) and SWI sequences (TE = 23.54 ms, TR = 45.40 ms, flip angle = 20°, matrix = 512 × 512, FOV = 240.64 × 240.64 mm^2^, 138 slices × 1 mm thickness) were used for CSVD assessment. WMH volume was quantified semi-automatically using the Wisconsin WMH Segmentation Toolbox, based on both T1-weighted and FLAIR images (24). DTI employed single-shot echo-planar imaging (SE-PI) with 64 diffusion gradient directions (*b*-value = 1,000 s/mm^2^) and 5 non-diffusion-weighted volumes (*b* = 0), using parameters: TR = 10,000 ms, TE = 74 ms, flip angle = 90°, matrix = 128 × 128, FOV = 256 × 256 mm^2^, 50 axial slices × 3 mm thickness. All images underwent comprehensive visual inspection for artifacts, structural abnormalities, and motion-related issues prior to analysis.

### 2.5 DTI data preprocessing

The DTI dataset was preprocessed using the FMRIB Software Library (FSL, http://www.fmrib.ox.ac.uk/fsl) (25). First, eddy current distortion and head motion were corrected by registering the diffusion-weighted images to the first b0 image through affine transformations (26). Second, non-brain tissue was removed using the FMRIB Brain Extraction Tool (BET) (27). FA and MD maps were generated using the DTIFIT toolbox. Third, FA maps were non-linearly registered to the Montreal Neurological Institute (MNI 152) standard space (28), skeletonized using a threshold of FA ≥ 0.2. MD maps underwent identical spatial transformations. Region of interest (ROI) analyses were conducted to extract mean FA/MD values from core WM tracts defined by the Johns Hopkins WM Tractography Atlas.

### 2.6 Evaluation of CSVD score

The overall CSVD score (range 0–7) integrated three neuroimaging markers: (1) WMH severity graded using the Fazekas scale (0–3) (29); (2) Lacunar infarct count on T2-weighted MRI; and (3) Presence/absence (0) of CMB (29, 30). Imaging assessments were independently conducted by two experienced neuroradiologists who were blinded to the clinical data. Discrepancies were resolved through consensus review, and inter-rater reliability was evaluated using Cohen's kappa coefficient (κ), which indicated substantial agreement.

### 2.7 Mediation analysis

To explore the potential role of WM impairment in the relationship between CSVD scores and cognitive function, mediation analysis was conducted using the PROCESS macro (http://www.processmacro.org/) (31). The analysis assessed the total effect (c) of CSVD score (X) on cognitive function (Y), the indirect effect (*a* × *b*) through WM fiber DTI index (*M*), and the direct effect (*c*') after accounting for the mediator (Supplementary Figure S1). Significance testing was performed using 5,000 bootstrap samples, with a significant indirect effect indicated if the bootstrap 95% confidence interval (CI) did not include 0. Only variables that exhibited significant correlations were considered as independent (CSVD score), dependent (cognitive function), or mediating variables (WM fiber DTI index) in the mediation analysis. Additionally, age, gender, years of education, and MetS components were included as covariates. A significance threshold of *P* < 0.05 was set.

### 2.8 Statistical analysis

The normality of the data was assessed using the Shapiro-Wilk test. Group differences in statistical and neuroimaging characteristics were analyzed using one-way analysis of variance (ANOVA) or the Kruskal–Wallis test for non-normally distributed data, and independent Student's *t*-test for normally distributed data. Categorical variables were analyzed using Pearson's chi-squared test. Multiple comparisons were corrected using Bonferroni correction for *post-hoc* group comparisons, the family-wise error rate (FWER) correction for FA and MD group comparisons, and the false discovery rate (FDR) correction for multiple linear regression analyses.

Group differences in FA and MD were analyzed using analysis of covariance (ANCOVA), adjusting for age, gender, and years of education. Partial correlation analysis was performed to investigate the relationship between neuropsychological characteristics and FA, controlling for the same covariates. Multiple linear regression analysis was used to examine the relationship between WM fiber DTI index (FA and MD) and cognitive function, with three models compared based on *R*^2^ and F statistics: Model 1 adjusted for age, gender, and years of education; Model 2 included these variables along with MetS components; and Model 3 incorporated the CSVD score. FDR correction was applied to account for multiple comparisons in these analyses. All analyses were performed using SPSS (version 25).

## 3 Results

### 3.1 Demographic characteristics and risk factors

This study included 170 participants: 75 with MetS and 95 HC. Demographic characteristics were well-matched between groups, with no significant differences in age, gender, or years of education (all *P* > 0.05, Table 1). As expected, the prevalence of hypertension and diabetes was significantly higher in the MetS group than in the HC group (*P* < 0.0001 for both). However, no significant intergroup differences were observed in smoking, drinking, or history of cardiovascular disease (all *P* > 0.05). As for MetS components, the MetS participants exhibited significantly higher values in WC, TG, Systolic blood pressure (SBP), diastolic blood pressure (DBP), and FBG compared to the HC (all *P* ≤ 0.0005), alongside significantly lower HDL-C levels (*P* < 0.0001). Additionally, CSVD scores were significantly elevated in the MetS group compared to the HC group (*P* < 0.0001). Among participants with MetS, those with CSVD (MetS-CSVD, *n* = 53) demonstrated significantly higher WC, SBP, DBP, and CSVD scores compared to those without CSVD (MetS-NCSVD, *n* = 22; *P* < 0.0001 for all).

**Table 1:** Demographic characteristics, MetS components, and CSVD scores of participants.

**Characteristics**	**HC (*N* = 95)**	**Subgroups of MetS**			***P*-value (HC vs. MetS-NCSVD)**	***P*-value (HC vs. MetS-CSVD)**	***P*-value (HC vs. MetS)**
		**MetS-NCSVD (*****N*** = **22)**	**MetS-CSVD (*****N*** = **53)**	***P*** **-value**			
**Baseline characteristics**							
Age (years, mean ± SD)	59.60 ± 7.80	58.95 ± 6.37	61.91 ± 5.27	0.095	0.694	0.054	0.183
Male (*n*, %)	53 (55.8)	12 (54.5)	30 (56.6)	0.870	0.916	0.924	0.978
Education (years, mean ± SD)	8.54 ± 3.08	7.36 ± 4.09	7.36 ± 4.09	0.816	0.186	0.139	0.079
**Vascular risk factors (** ***n*** **, %)**							
Smoking	20 (21.1)	6 (27.3)	12 (22.6)	0.669	0.527	0.822	0.647
Drinking	26 (27.4)	6 (27.3)	11 (20.8)	0.539	0.993	0.373	0.484
Hypertension	15 (15.8)	7 (31.8)	27 (50.9)	0.130	0.083	< 0.0001	< 0.0001
Diabetes	12 (12.6)	6 (27.3)	26 (49.1)	0.082	0.086	< 0.0001	< 0.0001
Cardiovascular disease	15 (15.8)	6 (27.3)	8 (15.1)	0.218	0.206	0.911	0.620
**MetS components (mean ±SD)**							
WC (cm)	80.98 ± 6.22	79.73 ± 8.20	87.02 ± 6.52	< 0.0001	0.424	< 0.0001	0.0003
HDL-C (mmol/L)	1.57 ± 0.35	1.24 ± 0.34	1.28 ± 0.44	0.673	0.0004	< 0.0001	< 0.0001
TG (mmol/L)	1.22 ± 0.70	1.94 ± 0.89	1.79 ± 1.10	0.493	0.001	0.0002	< 0.0001
SBP (mmHg)	125.02 ± 18.05	128 ± 22.55	150.7 ± 16.34	< 0.0001	0.490	< 0.0001	< 0.0001
DBP (mmHg)	77 ± 11.59	78.14 ± 13.66	93.09 ± 8.39	< 0.0001	0.663	< 0.0001	< 0.0001
FBG (mmol/L)	5.58 ± 0.64	6.29 ± 1.14	6.26 ± 1.92	0.910	0.017	0.002	0.0005
**Neuroimaging [median (Q1, Q3)]**							
CSVD score	0 (0, 1)	2.5 (2, 3)	5 (4, 6)	< 0.0001	< 0.0001	< 0.0001	< 0.0001

HC, healthy control; MetS-NCSVD, metabolic syndrome without cerebral small vessel disease; MetS-CSVD, metabolic syndrome with cerebral small vessel disease; WC, waist circumference; HDL-C, high density lipoprotein cholesterol; TG, triglyceride; SBP, systolic blood pressure; DBP, diastolic blood pressure, FBG, fasting blood glucose.

### 3.2 Cognitive function

Cognitive function was assessed using the MoCA, SCWT, and TMT. As shown in Table 2, participants with MetS exhibited significantly poorer cognitive performance compared to the HC group (*P* ≤ 0.001). Specifically, the MetS-CSVD group demonstrated the most severe cognitive impairments, with significant differences compared to the HC (*P* < 0.0001) and the MetS-NCSVD groups (*P* < 0.05).

**Table 2:** Cognitive function assessments across different groups.

**Cognitive tests**	**HC (*N* = 95)**	**Subgroups of MetS**			***P*-value (HC vs. MetS-NCSVD)**	***P*-value (HC vs. MetS-CSVD)**	***P*-value (HC vs. MetS)**
		**MetS-NCSVD (*N* = 22)**	**MetS-CSVD (*N* = 53)**	***P* **-value****			
MoCA (mean ± SD)	24.46 ± 2.98	22.41 ± 5.17	19.88 ± 5.78	0.023	0.046	< 0.0001	< 0.0001
SCWT-A (mean ± SD)	21.07 ± 7.63	21.24 ± 6.14	28.85 ± 15.41	0.005	0.947	< 0.0001	0.001
SCWT-B (mean ± SD)	24.39 ± 9.75	24.77 ± 5.52	34.47 ± 20.78	0.006	0.907	< 0.0001	0.001
SCWT-C (mean ± SD)	39.16 ± 15.99	32.69 ± 10.44	63.55 ± 21.07	< 0.0001	0.114	< 0.0001	< 0.0001
TMT-A (mean ± SD)	66.75 ± 31.76	68.39 ± 27.52	119.59 ± 76.72	0.0001	0.892	< 0.0001	< 0.0001
TMT-B (mean ± SD)	121.04 ± 57.86	116.22 ± 52.58	247.53 ± 106.22	< 0.0001	0.804	< 0.0001	< 0.0001
GDS [median (Q1, Q3)]	3 (1, 3)	3 (1, 3)	3 (2, 5)	0.162	0.732	0.075	0.213

HC, healthy control; MetS-NCSVD, metabolic syndrome without cerebral small vessel disease; MetS-CSVD, metabolic syndrome with cerebral small vessel disease; MoCA, Montreal cognitive assessment; SCWT, Stroop color word test; TMT, trail making test; GDS, geriatric depression scale.

### 3.3 WM microstructural alterations

**Figure 1:**
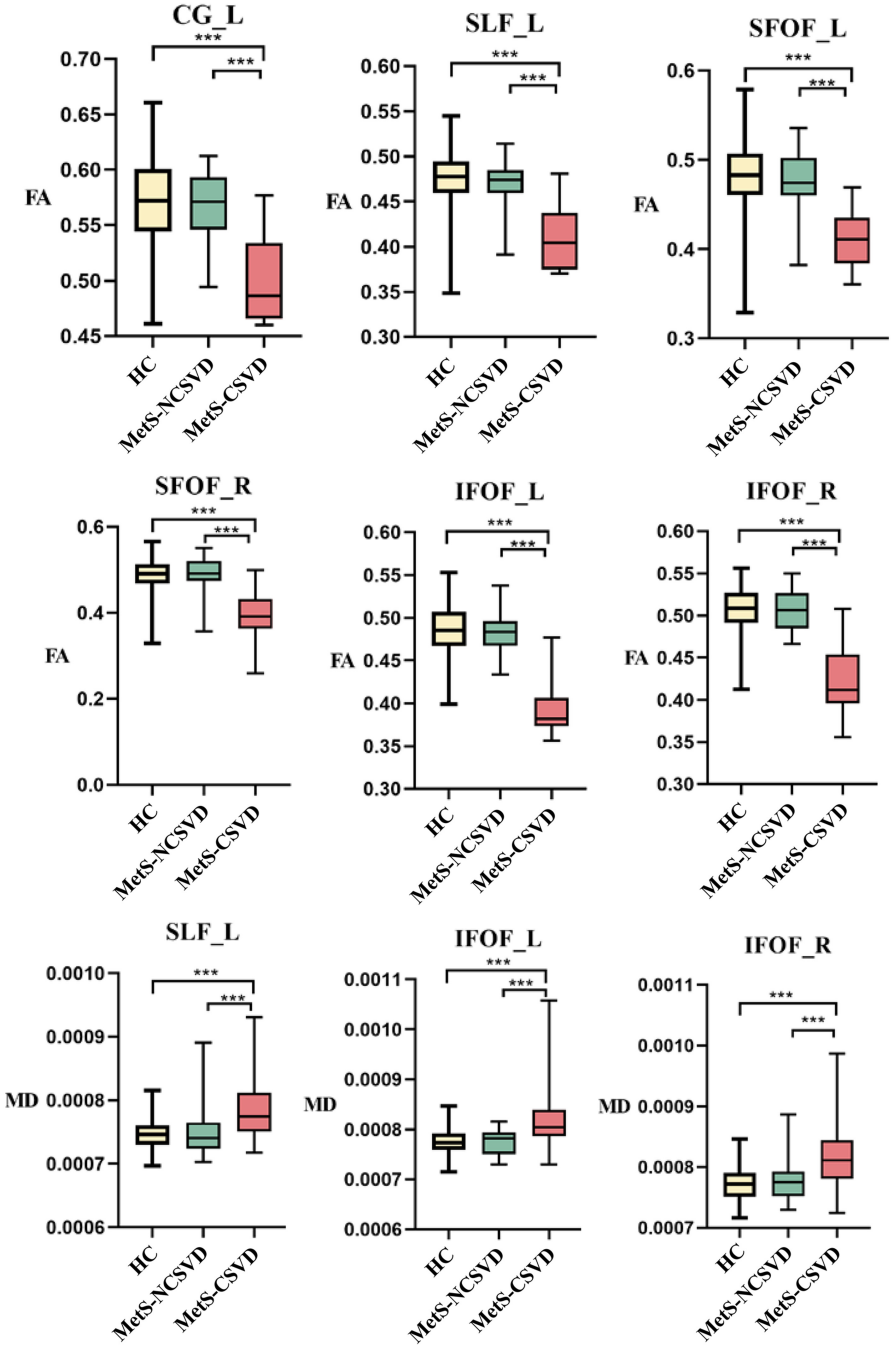
Fractional anisotropy (FA) and mean diffusivity (MD) in white matter fiber tracts across different groups. HC, healthy control; MetS-NCSVD, metabolic syndrome without cerebral small vessel disease; MetS-CSVD, metabolic syndrome with cerebral small vessel disease; CG, cingulum; SLF, superior longitudinal fasciculus; SFOF, superior fronto-occipital fasciculus; IFOF, inferior fronto-occipital fasciculus; L, left; R, right. ***P < 0.001.

The microstructural integrity of WM fiber tracts was assessed using FA and MD values. In the MetS-CSVD group, FA values were significantly lower compared to the MetS-NCSVD and HC groups in several core WM fiber tracts, including the left cingulum (CG_L), left superior longitudinal fasciculus (SLF_L), left superior fronto-occipital fasciculus (SFOF_L), right superior fronto-occipital fasciculus (SFOF_R), left inferior fronto-occipital fasciculus (IFOF_L), and right inferior fronto-occipital fasciculus (IFOF_R) (*P* < 0.001, corrected for FWER) (Figure 1). Additionally, the MD values were significantly higher in the MetS-CSVD group in SLF_L, IFOF_L, and IFOF_R (*P* < 0.001, corrected for FWER) (Figure 1).

### 3.4 Associations between WM microstructural alterations and cognitive function

**Figure 2:**
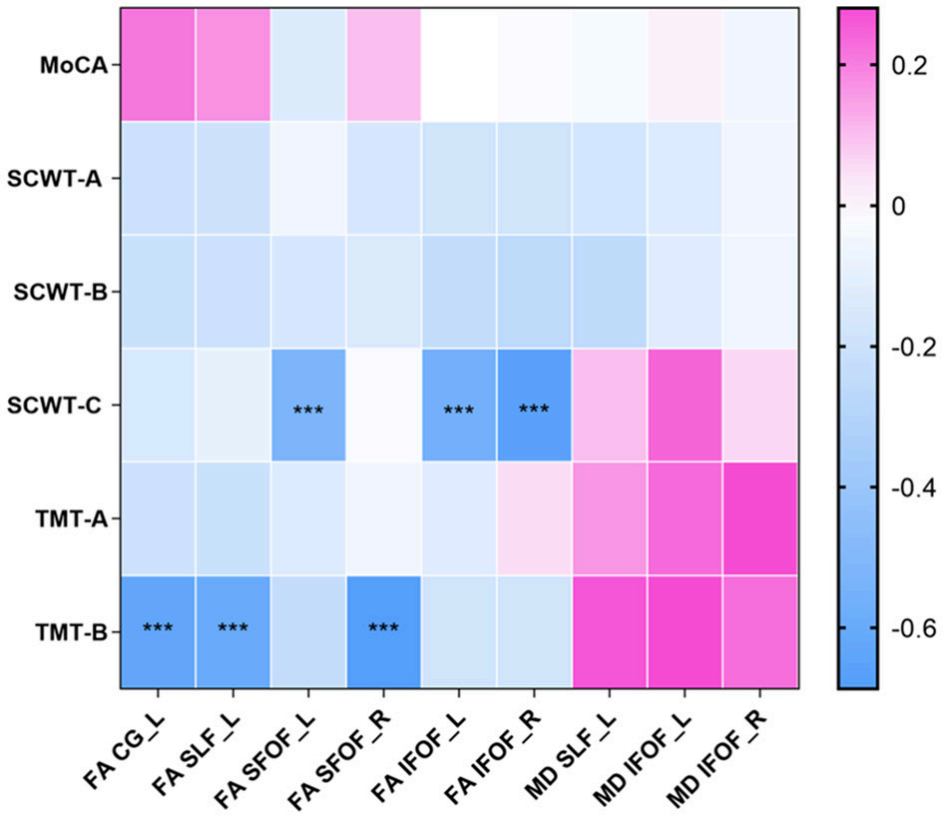
Heatmap of the associations between white matter microstructural alterations and cognitive function in the MetS-CSVD group. MoCA, Montreal cognitive assessment; TMT, trail making test; SCWT, Stroop color and word test; FA, fractional anisotropy; MD, mean diffusivity; CG, cingulate gyrus; SLF, superior longitudinal fasciculus; SFOF, superior frontal-occipital fasciculus; IFOF, inferior frontal-occipital fasciculus; R, right; L, left. ***P < 0.001.

**Figure 3:**
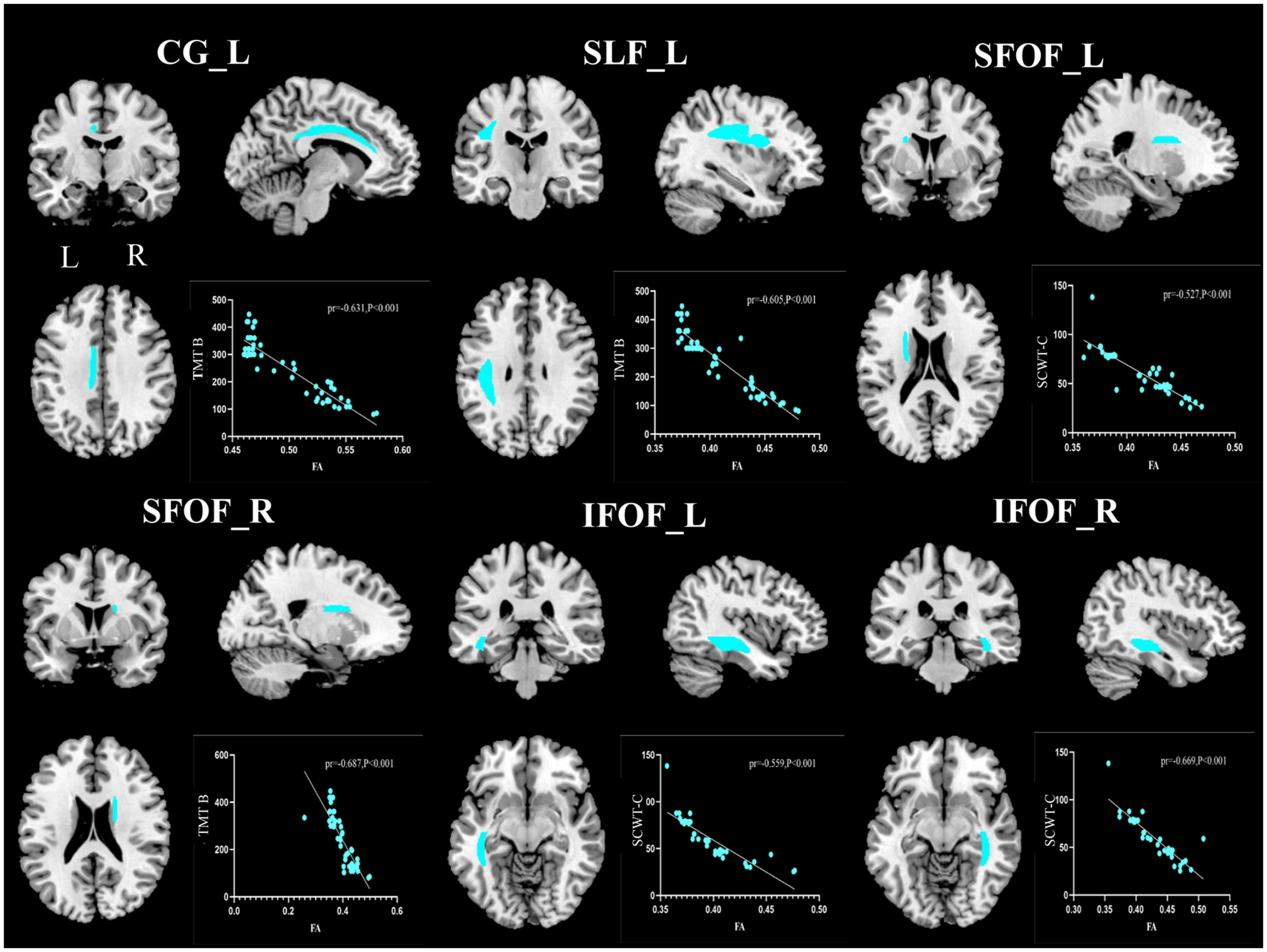
Partial correlation between fractional anisotropy (FA) of white matter fiber tracts and cognitive function. CG, cingulum; SLF, superior longitudinal fasciculus; SFOF, superior fronto-occipital fasciculus; IFOF, inferior fronto-occipital fasciculus; L, left; R, right. pr denotes the partial correlation coefficient.

The relationship between WM microstructural alterations and cognitive function was examined in the MetS-CSVD group (Figure 2). After adjusting for age, gender, and years of education, significant inverse relationships were observed between certain cognitive functions and FA values of several core WM fiber tracts. Specifically, TMT-B scores exhibited significant negative correlations with FA values in the CG_L (pr = −0.631, *P* < 0.001), SLF_L (pr = −0.605, *P* < 0.001), and SFOF_R (pr = −0.687, *P* < 0.001) (Figures 2, 3). Similarly, the SCWT-C scores were inversely correlated with FA values in the SFOF_L (pr = −0.527, *P* < 0.001), IFOF_L (pr = −0.559, *P* < 0.001) and IFOF_R (pr = −0.669, *P* < 0.001) (Figures 2, 3).

To further validate these associations, multiple linear regression analyses were conducted. The associations between FA values in core WM fiber tracts and cognitive function were consistent across Model 1 and Model 2. Specifically, SCWT-C scores were negatively associated with FA values in the SFOF_L (β = −202.607, *P* = 0.027), IFOF_L (β = −280.311, *P* < 0.001), and IFOF_R (β = −212.281, *P* = 0.012) (Table 3). Similarly, TMT-B scores were negatively associated with FA values of CG_L (β = −1077.744, *P* = 0.045), SLF_L (β = −1216.599, *P* = 0.019), and SFOF_R (β = −570.307, *P* = 0.032) (Table 4). Furthermore, when the CSVD score was incorporated into Model 3, the CSVD score itself emerged as a significant independent predictor of worse cognitive performance (β = 4.073, *P* = 0.009 for SCWT-C; β = 12.244, *P* = 0.004 for TMT-B).

**Table 3:** Relationship between Stroop color word test (SCWT)-C and fractional anisotropy of several core WM fiber tracts in the MetS-CSVD group.

**Models**	**Variables**	**SFOF_L**	**IFOF_L**	**IFOF_R**	**CSVD score**	***R* ^2^**
Model-1	B	−202.607	−280.311	−212.281		0.864 (*F* = 111.07, *P* < 0.001)
	SE	88.670	72.761	80.916		
	β	−0.284	−0.358	−0.345		
	t	−2.285	−3.853	−2.623		
	p	0.027	< 0.001	0.012		
Model-2	B	−202.607	−280.311	−212.281		0.864 (*F* = 111.07, *P* < 0.001)
	SE	88.670	72.761	80.916		
	β	−0.284	−0.358	−0.345		
	t	−2.285	−3.853	−2.623		
	p	0.027	< 0.001	0.012		
Model-3	B		−205.883	−284.465	4.073	0.869 (*F* =116.22, *P* < 0.001)
	SE		80.750	59.181	1.496	
	β		−0.263	−0.462	0.270	
	t		−2.550	−4.807	2.723	
	p		0.014	< 0.001	0.009	

Model 1 adjusted for age, gender, and years of education; Model 2 adjusted for age, gender, years of education, systolic blood pressure (SBP), diastolic blood pressure (DBP), triglycerides (TG), high-density lipoprotein cholesterol (HDL-C), waist circumference (WC), fasting blood glucose (FBG); Model 3 adjusted for age, gender, years of education, SBP, DBP, TG, HDL-C, WC, FBG and CSVD score. SFOF, superior fronto-occipital fasciculus; IFOF, inferior fronto-occipital fasciculus; L, left; R, right; B, regression coefficient; SE, standard error; β, standardized coefficient.

**Table 4:** Relationship between trail making test (TMT)-B and fractional anisotropy of several core WM fiber tracts in the MetS-CSVD group.

**Models**	**Variables**	**CG_L**	**SLF_L**	**SFOF_R**	**CSVD score**	***R* ^2^**
Model-1	B	−1077.744	−1216.599	−570.307		0.855 (*F* = 103.20, *P* < 0.001)
	SE	524.078	500.931	257.731		
	β	−0.369	−0.367	−0.232		
	*t*	−2.056	−2.429	−2.213		
	*p*	0.045	0.019	0.032		
Model-2	B	−1077.744	−1216.599	−570.307		0.855 (*F* = 103.20, *P* < 0.001)
	SE	524.078	500.931	257.731		
	β	−0.369	−0.367	−0.232		
	*t*	−2.056	−2.429	−2.213		
	*p*	0.045	0.019	0.032		
Model-3	B		−1848.948	−899.556	12.244	0.867 (*F* = 114.44, *P* < 0.001)
	SE		287.703	207.377	4.03	
	β		−0.557	−0.367	0.161	
	*t*		−6.427	−4.338	3.039	
	*p*		< 0.001	< 0.001	0.004	

Model 1 adjusted for age, gender, and years of education; Model 2 adjusted for age, gender, years of education, systolic blood pressure (SBP), diastolic blood pressure (DBP), triglycerides (TG), high-density lipoprotein cholesterol (HDL-C), waist circumference (WC), fasting blood glucose (FBG); Model 3 adjusted for age, gender, years of education, SBP, DBP, TG, HDL-C, WC, FBG and CSVD score. CG, cingulum; SLF, superior longitudinal fasciculus; SFOF, superior fronto-occipital fasciculus; L, left; R, right; *B*, regression coefficient; SE, standard error; β, standardized coefficient.

### 3.5 Mediation analysis

**Figure 4:**
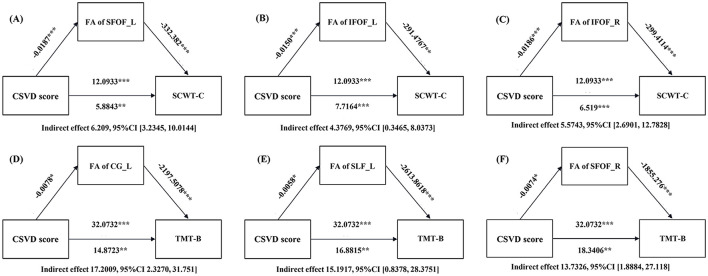
Mediation analysis of the relationship between CSVD score, white matter fiber DTI index, and cognitive function. **(A)** Mediation model for fractional anisotropy (FA) of the left superior fronto-occipital fasciculus (SFOF_L) between CSVD score and the Stroop color word test (SCWT)-C. **(B)** Mediation model for FA of the left inferior fronto-occipital fasciculus (IFOF_L) between CSVD score and SCWT-C. **(C)** Mediation model for FA of the right inferior fronto-occipital fasciculus (IFOF_R) between CSVD score and SCWT-C. **(D)** Mediation model for FA of the left cingulum (CG_L) between CSVD score and the trail making test (TMT)-B. **(E)** Mediation model for FA of the left superior longitudinal fasciculus (SLF_L) between CSVD score and TMT-B. **(F)** Mediation model for FA of the right superior fronto-occipital fasciculus (SFOF_R) between CSVD score and TMT-B. All analyses were adjusted for age, gender, years of education, waist circumference (WC), high-density lipoprotein cholesterol (HDL-C), triglycerides (TG), systolic blood pressure (SBP), diastolic blood pressure (DBP), and fasting blood glucose (FBG). *P < 0.05, **P < 0.01, ***P < 0.001.

To further explore whether the WM microstructural alterations, as indicated by FA values, mediated the effect of CSVD severity on cognitive function, a mediation analysis was conducted. Results showed that there were significant indirect effects of CSVD score on cognitive test scores through FA values in core WM fiber tracts. Specifically, FA values in the SFOF_L (indirect effect = 6.209, 95% CI = 3.2345 to 10.0144, Figure 4A), IFOF_L (indirect effect = 4.3769, 95% CI = 0.3465 to 8.0373, Figure 4B), and IFOF_R (indirect effect = 5.5743, 95% CI = 2.6901 to 12.7828, Figure 4C) mediated the relationship between CSVD score and SCWT-C. Additionally, FA value in the CG_L (indirect effect = 17.2009, 95% CI = 2.3270 to 31.751, Figure 4D), SLF_L (indirect effect = 15.1917, 95% CI = 0.8378 to 28.3751, Figure 4E), and IFOF_R (indirect effect = 13.7326, 95% CI = 1.8884 to 27.118, Figure 4F) mediated the relationship between CSVD score and TMT-B.

## 4 Discussion

MetS is a multifactorial condition that has garnered significant attention due to its profound impact on public health (32). It is associated with an increased risk of cardiovascular disease and cognitive impairment (33). Our findings are consistent with previous research, indicating that participants with MetS exhibit significant cognitive decline. Each component of MetS may affect brain structure and function through distinct mechanisms (34). For instance, hypertension can lead to endothelial dysfunction, thereby impairing cerebral blood flow (35). Hyperglycemia may damage neurons through oxidative stress and inflammatory responses (36, 37). These processes are associated with microstructural changes in WM fibers, which may subsequently influence cognitive function.

Although CSVD is recognized as a key pathological link between MetS and cognitive impairment (38), the specific pathways through which MetS contributes to CSVD and subsequent cognitive deficits are not fully elucidated. In this study, we found that cognitive function in the MetS-CSVD group was significantly negatively correlated with FA values of WM fibers. Meanwhile, the MetS-CSVD group exhibited lower FA values in multiple brain regions, including the CG_L, SLF_L, SFOF_L, SFOF_R, IFOF_L, and IFOF_R, compared to the MetS-NCSVD and HC groups. Reduced FA values, a key indicator of WM fiber microstructural integrity, may imply axonal damage, myelin loss, or decreased fiber density (39). Such WM fiber damage is hypothesized to impair the efficiency of information transfer between different brain regions, thereby potentially contributing to cognitive impairment. Furthermore, the MetS-CSVD group had higher MD values in the SLF_L, IFOF_L, and IFOF_R. Elevated MD values are typically associated with tissue damage or inflammation, which may further exacerbate the damage to WM fibers, disrupt their normal function, and consequently have a negative impact on cognitive function (40).

WM fiber damage not only reduces the efficiency of information transfer but also may lead to cognitive impairments by impacting the function of specific cognitive networks, particularly in executive function and information processing speed (41–43). For instance, damage to WM fibers in regions such as CG_L and SLF_L may affect executive function and attention (41). The IFOF connects networks of cognitive control, attention, language processing, and working memory, and its damage to IFOF fibers has been linked to impairments in these functions (42). The SLF and IFOF fiber bundles influence the hippocampal-prefrontal circuit, and disruptions in these pathways may contribute to cognitive difficulties in patients (44, 45). Thus, our findings suggest that the degradation of these specific tracts may impair the integrity of large-scale neurocognitive networks, such as the frontoparietal and limbic systems, thereby identifying a potential neurobiological mechanism for the executive deficits in MetS-CSVD.

A study demonstrated a direct relationship between elevated SBP and reduced FA, as well as increased MD, in specific brain regions, including the IFOF and pathways connecting the superior frontal gyrus to the thalamus (46). The bilateral SFOF plays a key role in executive function by linking the parietal lobe to the dorsolateral prefrontal cortex (47). In this study, patients with MetS who exhibited lower FA values in the SFOF also demonstrated poorer cognitive function, which is consistent with the observed association between MetS-related WM alterations and cognitive deficits. Multiple studies have reported a higher likelihood of dementia in participants with MetS compared to those without the syndrome (48–50).

Our findings indicate that patients with higher CSVD scores exhibited poorer cognitive function, with the most pronounced deficits observed in the MetS-CSVD group compared to the MetS-NCSVD and HC groups. Although CSVD is recognized as the primary vascular contributor to cognitive impairment and dementia (51), the precise mechanisms underlying this association remain unclear. The multiple regression model in this study demonstrated that higher CSVD scores were significantly and independently associated with worse cognitive function. This suggests that CSVD may be integrally involved in the pathway to cognitive impairment in participants with MetS. Accumulating evidence indicates that CSVD exerts its clinical effects by disrupting WM connectivity (52), as shown by cross-sectional studies linking cognitive impairment to diffuse brain damage and lacunes detected through DTI (53). These structural alterations contribute to WM pathway disruption and disconnection of distributed networks, predominantly affecting processing speed and executive function, two cognitive domains that are particularly vulnerable to CSVD-related changes (52, 54). Cognitive impairment has been associated with WM microstructural damage, with one hypothesis proposing that impairments in WM pathways and subsequent disruptions in complex networks linking the cerebral cortex and subcortical regions contribute to cognitive dysfunction (55, 56).

Our findings suggest that WM microstructural damage plays a significant role in the cognitive impairment observed in participants with MetS-CSVD, with alterations in core WM fiber tracts mediating the association between CSVD severity and cognitive function. Specifically, cognitive function, as assessed by the SCWT-C and TMT-B, was significantly correlated with FA reductions in core WM fiber tracts, including the CG_L, SLF_L, and IFOF_R. These tests primarily evaluate executive function, cognitive flexibility, and processing speed, cognitive domains that are particularly vulnerable to CSVD-related WM disruption (57).

Our mediation analysis further supports this interpretation, demonstrating that FA reductions in core WM fiber tracts partially explain the link between CSVD severity and cognitive impairment. Notably, FA in the SFOF_L and IFOF_L mediated the relationship between CSVD scores and SCWT-C scores, while FA of the CG_L, SLF_L, and IFOF_R mediated the relationship between CSVD scores and TMT-B scores. These findings suggest that WM microstructural alterations serve as a mechanistic pathway through CSVD which pathology contributes to cognitive dysfunction, which is consistent with previous studies (58). Specifically, the observed FA reductions in these tracts are not merely imaging biomarkers but likely reflect underlying pathologies, which can be driven by CSVD-related chronic hypoperfusion and blood-brain barrier disruption (21). The metabolic derangements in MetS may exacerbate these processes by promoting endothelial dysfunction and neuroinflammation, thereby accelerating the disintegration of WM structural networks. Given that executive function and processing speed rely on the integrity of large-scale fronto-subcortical networks, these results reinforce the notion that CSVD leads to disrupted connectivity, ultimately impairing cognitive efficiency (59).

This study has several limitations. First, the small sample size and uneven group distribution may influence the generalizability and statistical power of our findings. To mitigate this concern, we employed appropriate statistical approaches, including non-parametric tests and FDR correction. Our primary findings focus on the more populous MetS-CSVD group. Nevertheless, conclusions regarding the MetS-NCSVD subgroup should be interpreted with caution. Second, we treated MetS as a unified entity and did not assess potential heterogeneity in WM injury across different MetS phenotypic presentations. Third, the cross-sectional design limits our ability to make causal inferences about the relationships among MetS, CSVD, and cognitive impairment. Additionally, the lack of longitudinal follow-up makes it challenging to determine whether CSVD and cognitive impairment are distinct outcomes or part of a progressive pathway. Finally, DTI limitations include the inability of the single-tensor model to resolve crossing fibers, potential residual motion artifacts despite correction, and the non-specific nature of FA and MD to underlying pathology. Future studies should aim for larger and more balanced sample sizes to enhance robustness. Such cohorts would also enable the investigation of whether specific MetS components drive distinct patterns of WM damage and cognitive decline. Moreover, employing more specific DTI metrics, such as axial and radial diffusivity, could help delineate the relative contributions of axonal damage vs. demyelination to the observed microstructural decline. Longitudinal designs could clarify temporal relationships and the sequence of conditions. Including a pure CSVD population without MetS could also help disentangle the unique contributions of MetS. Addressing these limitations will deepen our understanding and aid in developing targeted interventions.

## 5 Conclusions

This study provides preliminary, cross-sectional evidence that WM microstructural damage, as indicated by reduced FA, may mediate the relationship between CSVD severity and cognitive impairment in patients with MetS. These findings potentially highlight WM integrity as a pathway through which CSVD is linked to cognitive impairment in MetS. Further longitudinal studies are needed to validate these observed relationships, and additional mechanistic research on metabolic dysregulation and neuroinflammation may help to better understand the underlying mechanisms and identify potential therapeutic targets.

## Data Availability

The original contributions presented in the study are included in the article/Supplementary material, further inquiries can be directed to the corresponding author.

## Supplementary material

Supplementary Figure S1 Mediation model of the relationship between CSVD score (*X*), WM fiber DTI index (*M*), and cognitive function (*Y*). The total effect (*c*) of the CSVD score (X) on cognitive function (*Y*) is decomposed into the direct effect (*c*') and the indirect effect (*a* × *b*) mediated by the WM fiber DTI index (*M*).
