# Supplementary figures and images for: White matter microstructural damage and its effect on cognitive impairment in patients with metabolic syndrome and cerebral small vessel disease
Source: Front Neurol. 2026 Jan 16;16:1698280. doi: 10.3389/fneur.2025.1698280 (PMC12855072; doi:10.3389/fneur.2025.1698280)

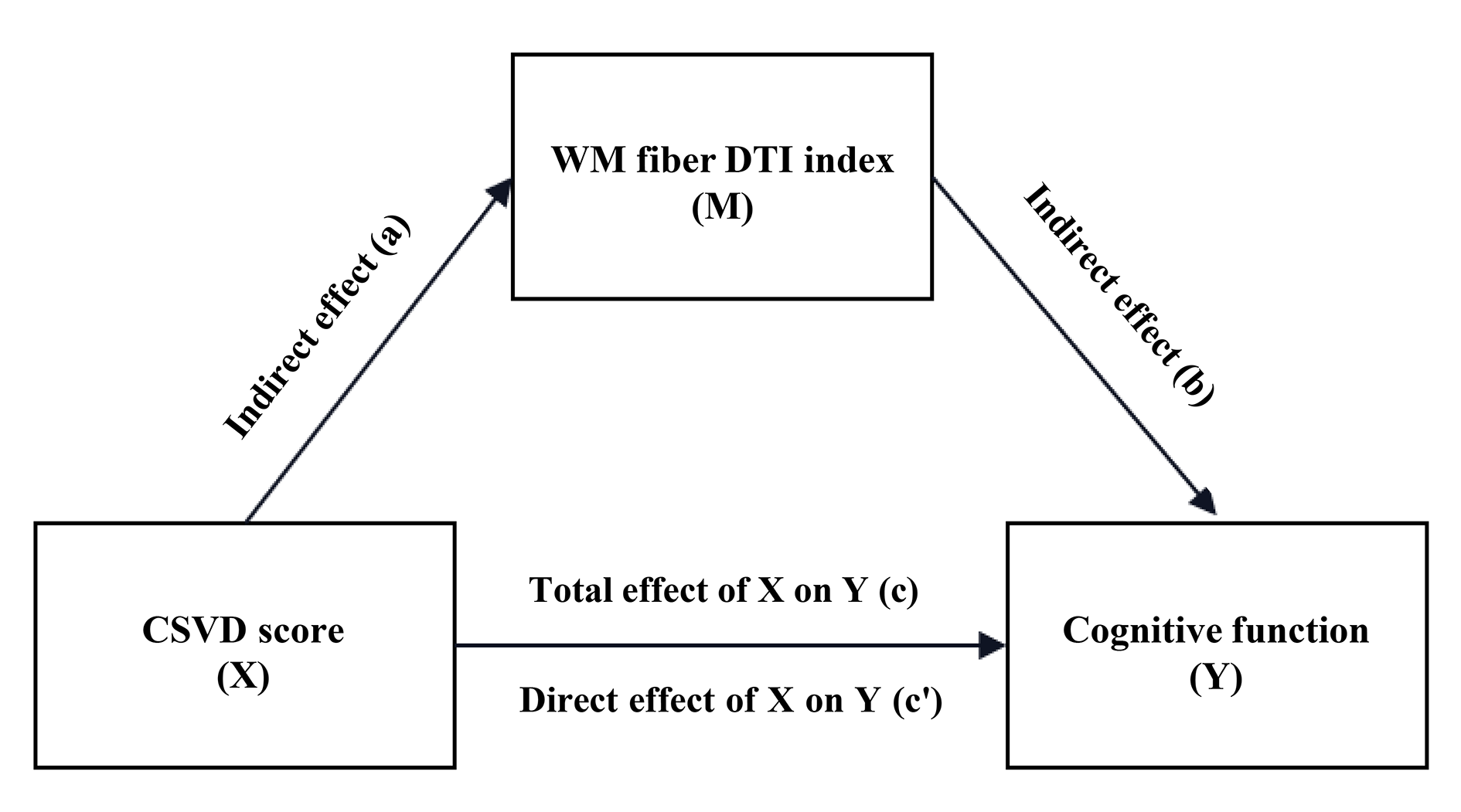

Supplement: Supplementary Figure S1 — Mediation model of the relationship between CSVD score (X), WM fiber DTI index (M), and cognitive function (Y). The total effect (c) of the CSVD score (X) on cognitive function (Y) is decomposed into the direct effect (c') and the indirect effect (a × b) mediated by the WM fiber DTI index (M). [file Image_1.tif]
